# Tim-3 Is Differentially Expressed during Cell Activation and Interacts with the LSP-1 Protein in Human Macrophages

**DOI:** 10.1155/2023/3577334

**Published:** 2023-10-26

**Authors:** Ranferi Ocaña-Guzman, Lucero A. Ramon-Luing, Luis A. Vazquez-Bolaños, Michelle Rodríguez-Alvarado, Fausi Bulhusen-Rodriguez, Alonso Torres-Hatem, Karen Gonzalez-Torres, Mariana Citlalli de Alba-Alvarado, Isabel Sada-Ovalle

**Affiliations:** ^1^Laboratory of Integrative Immunology, Instituto Nacional de Enfermedades Respiratorias “Ismael Cosío Villegas”, Mexico City, Mexico; ^2^Posgrado en Ciencias Biológicas, Universidad Nacional Autónoma de México, Mexico; ^3^Department of Microbiology and Parasitology, Faculty of Medicine, National Autonomous University of Mexico, Coyoacán, México City 04510, Mexico; ^4^Physiology Department, Medicine School Universidad Autónoma de San Luis Potosí, San Luis Potosí, Mexico

## Abstract

T-cell Immunoglobulin and Mucin Domain 3 (TIM-3) is an immune checkpoint receptor known to regulate T-cell activation and has been targeted for immunotherapy in cancer and other diseases. However, its expression and function in other cell types, such as macrophages, are poorly understood. This study investigated TIM-3 expression in human macrophages polarized to M1 (stimulated with IFN-*γ* and LPS) and M2 (stimulated with IL-4 and IL-13) phenotypes using an in vitro model. Our results show that M1 macrophages have a lower frequency of TIM-3+ cells compared to M2 macrophages at 48 and 72 hr poststimulation. Additionally, we observed differential levels of soluble ADAM 10, an enzyme responsible for TIM-3 release, in the supernatants of M1 and M2 macrophages at 72 hr. We also found that the TIM-3 intracellular tail might associate with lymphocyte-specific protein 1 (LSP-1), a protein implicated in cell motility and podosome formation. These findings enhance our understanding of TIM-3 function in myeloid cells such as macrophages and may inform the development of immunotherapies with reduced immune-related adverse effects.

## 1. Introduction

Macrophage activation is a multifaceted process coordinating various biochemical mechanisms, encompassing cytoplasmic proteins, enzymes, and membrane receptors. This activation enables macrophages to adopt a function-specific profile in response to the microenvironment and the unique signals detected in specific tissues, a phenomenon called macrophage plasticity [1].

To initiate activation and polarization, macrophages must recognize non-self-molecules, such as pathogen-associated molecular patterns (PAMPs) or damage-associated molecular patterns (DAMPs). A diverse array of receptors, including toll-like receptors (TLRs), nucleotide-binding oligomerization domain-like receptors (NLRs), retinoic acid-inducible gene-I- (RIG-I-) like receptors (RLRs), and scavenger receptor families, mediates the recognition of PAMPs and DAMPs, subsequently inducing activation and guiding macrophage polarization. While specific receptors facilitate signaling pathways for macrophage activation, others function to regulate this process to prevent an excessive or unnecessary response due to chronic activation. For example, receptors such as TIM-3, PD-1, CD32b, and CD200R can inhibit or modulate macrophage activation. To avoid an overactive response by innate immunity cells, this activation process necessitates careful regulation, which can be achieved by inhibitory receptors, also known as immune checkpoints [2–6].

TIM-3 is an inhibitory receptor predominantly expressed by lymphoid cells, particularly TH1 lymphocytes [7–9]. Chronically activated lymphocytes have been observed to overexpress this receptor [10, 11]. Sustained cell activation has been linked to the prolonged expression of inhibitory receptors, resulting in a characteristic T-cell exhaustion [12]. Such exhausted T cells exhibit a diminished capacity to produce cytokines and proliferate in response to antigenic stimuli.

In addition to T cells, TIM-3 has been implicated in the modulation of macrophage activation; however, its physiological or pathophysiological role in macrophages is diverse and not fully understood [13–16]. The mechanisms by which TIM-3 work in these cells warrant further investigation.

Evidence shows that TIM-3 expression and function are dynamic in monocytes and macrophages. In a mouse model of liver cancer, tumor-associated macrophages (TAMs) displayed increased TIM-3 expression, likely due to the presence of transforming growth factor-*β* (TGF-*β*) that mediates TIM-3 expression [16]. TAMs exhibit an M2-like profile characterized by the expression of CD163, CD206, Arginase-1, and IL-10. Moreover, they lose or reduce many protective functions in cancer, thus promoting tumor development and progression [16]. In contrast, Ma et al. [17] demonstrated that TIM-3 expression on macrophages decreases rapidly (within 6 hr) upon TLR stimulation; conversely, the interaction of TIM-3/GAL-9 negatively regulates TLR signaling, leading to a reduction in IL-12 production and an increase in IL-23 production.

Blocking TIM-3 with monoclonal antibodies before TLR ligand stimulation has been shown to increase the production of IL-12, IL-6, and IL-10 [17–19]. These findings indicate that IL-12 expression is negatively regulated by TIM-3, which is highly expressed on resting macrophages but decreases following TLR stimulation. Further studies have demonstrated that TIM-3 also modifies macrophage functionality based on their specific tasks. For instance, TIM-3 mediates the uptake and phagocytosis of apoptotic bodies in a phosphatidylserine-dependent pathway [20]. Likewise, TIM-3 has been implicated in regulating the NLRP3 inflammasome [21, 22] and indirectly controlling IL-1*β* production. Supporting these findings, in vivo administration of TIM-3 mAb was shown to enhance the clinical and pathological severity of experimental autoimmune encephalomyelitis (EAE), an effect associated with an increased number of pro-inflammatory (M1) macrophages [23]. In summary, evidence suggests that TIM-3 modulates macrophage activation depending on their functionality; while M2 macrophages overexpress TIM-3, M1 macrophages exhibit low TIM-3 expression.

Current knowledge regarding the dynamics of TIM-3 expression during macrophage polarization remains incomplete. Therefore, in this study, we aimed to investigate the dynamics of TIM-3 expression in M1 and M2 macrophages.

## 2. Materials and Methods

### 2.1. Cells

Peripheral blood mononuclear cells (PBMCs) were isolated from buffy coats of healthy donors at the blood bank of the National Institute of Respiratory Diseases in Mexico City. PBMCs were collected and counted to determine their viability using the TC20 cell counter system (Bio-Rad, California, USA). Monocytes were enriched by employing positive selection with magnetic microbeads coated with CD14 antibody (Miltenyi Biotech, Bergisch Gladbach, Germany). The purity was confirmed by flow cytometry, and the purified cells were found to be 90%–95% of the intended cell type.

### 2.2. Cell Culture

Monocytes were plated at 1 × 10^6^ cells per well in 12-well plates (Costar, ON, Canada) with RPMI-1640 medium supplemented with 10% FBS (Gibco BRL, Grand Island, NY, USA). After a 7-day incubation period (37°C, 5% CO_2_), viable cells were considered human monocyte-derived macrophages (MDMs) based on their expression profile of CD14, CD16, CD68, and HLA-DR. On the 7th day, macrophage polarization was induced by removing the culture medium and culturing cells for 72 hr in RPMI-1640 supplemented with IFN-*γ* 20 ng/mL + LPS 50 ng/mL (for M1 polarization) or IL-4 20 ng/mL + IL-13 20 ng/mL (for M2 polarization) [3, 23]. Additional IL-4 was added every 24 hr from day four following the initial stimuli. CD14+ cells were seeded and cultured at 37°C in a humidified atmosphere containing 5% CO_2_ for 7 days. Following this period, viable cells with distinct morphology and cell surface expression profiles were considered MDM.

### 2.3. Flow Cytometry

TIM-3, BAT-3, and GAL-9 were evaluated by multiparametric flow cytometry. MDMs were collected and stained for 20 min at 4°C with monoclonal antibodies (mAb) to CD14, CD68, and GAL-9. TIM-3 (BioLegend) was labeled with two mAb (Clone F38–2E2, BrilliantViolet421); first, staining was performed to saturate surface TIM-3 epitopes. Next, fixation/permeabilization procedures were performed, followed by staining of intracytoplasmic TIM-3 epitopes using the same clone of APC-conjugated TIM-3 mAb (F38–2E2) and subsequent flow cytometric analysis. The Zombie Aqua Fixable Viability Kit (BioLegend) was included to evaluate cell viability. A FACS Aria II Flow cytometer (Becton Dickinson, San Jose, CA) was used for acquisition and compensation with a single fluorochrome. Data were analyzed using FlowJo software V.10.0.8. Cells used for FMO conditions were stained and acquired in parallel. Dead cells were excluded using the side scatter/forward gating strategy and Zombie Aqua exclusion (BioLegend), and isotype-matched control antibodies were used to identify background staining levels. Typically, 50,000 events were recorded. Antibodies were used following the manufacturer's recommended dilution or established by titration assays. All antibodies used in this study are listed in Table S1.

### 2.4. RNA Extraction and RT-PCR

Following IFN-*γ*/LPS (M1) or IL-4/IL-13 (M2) stimulation, activated macrophages were lysed at 0, 24, 48, and 72 hr. RNA was extracted according to the manufacturer's instructions using the RNeasy Mini Kit (Qiagen, Hilden, Germany). Genomic DNA contamination was removed with the RNA-Free DNase Set (Qiagen), and nucleic acid quantification was conducted using a Qubit 2.0 fluorometer (Life Technologies, Waltham, MA, USA). For cDNA synthesis, 30 ng/*μ*L of RNA was utilized for RT-PCR with the High-capacity cDNA Reverse Transcription Kit (Applied Biosystems, Waltham, MA, USA). Gene expression was evaluated by quantitative real-time PCR (qPCR) and analyzed using the *ΔΔ*Ct method with TaqMan probes targeting TIM3 (Hs00958618_m1), GAL9 (Hs01088490_m1), BAT3 (Hs00190383_m1), and ADAM10 (Hs00153853_m1) genes. Concurrently, 18S ribosomal RNA gene (Hs03928990_g1) and ACTB (*β*-actin) (Hs01060665_g1) were employed as endogenous controls. Individual reactions were prepared using the Maxima Probe/ROX qPCR Master Mix (Thermo Fisher Scientific, Waltham, MA, USA) and run under the following thermal cycling conditions: 95°C for 10 min, followed by 40 cycles of 60°C for 1 min and 95°C for 15 s in the Step One Plus Real-Time PCR System (Applied Biosystems, Waltham, MA, USA). Unstimulated macrophages served as the reference condition (where RQ = 2^−*ΔΔ*^CT = 1).

### 2.5. Immunoprecipitation and Western Blot

After removing the medium, 1 × 10^6^ macrophages were washed with phosphate-buffered saline (PBS) and incubated for 15 min at 4°C with Pierce IP lysis buffer (Thermo Scientific) containing the protease inhibitor Cocktail P8340 (Sigma–Aldrich). Lysates were collected and centrifuged at 13,000 *g* for 10 min to remove membrane debris. Immunoprecipitation (IP) was carried out using the Dynabeads Protein G Kit (Invitrogen) and 1 *μ*g of monoclonal antibody specific for TIM-3 (Biolegend). IP was conducted according to the manufacturer's instructions, and the precipitated proteins were analyzed by electrophoresis in 10% SDS-PAGE under reducing conditions, followed by transfer to nitrocellulose using standard procedures. BAT-3 (R&D Systems), LSP-1(Cell Signaling Technology), were detected using 1 *μ*g of specific monoclonal antibodies or the manufacturer-recommended dilution.

### 2.6. Galectin-9 Treatment

MDM obtained from 5 × 10^5^ monocytes were collected and washed three times with PBS. Subsequently, cells were incubated with 1 mL of RPMI containing GAL-9 (50 nM) at 10 min. After incubation, cell pellets and culture supernatants were collected. Cells were then lysed with IP Lysis buffer (Thermo Fisher), and the lysates were used for TIM-3 immunoprecipitation, as described previously. The culture supernatants from activated macrophages were stored at −80°C until further use.

### 2.7. ELISA

Soluble molecules of interest were assessed in supernatants (SN) obtained from macrophage cell cultures. SNs were stored at −20°C until analysis. Standard sandwich ELISA was performed for IL-1*β* (BioLegend Cat. 437015) and IL-10 cytokines (BioLegend, Cat. 430604) according to the manufacturer's instructions. The ADAM-10 proenzyme ELISA kit (Cat. SEA766Hu) was provided by Cloud-Clone Corp. (USCN) and used as directed.

### 2.8. Protein Identification by Peptide Mass Fingerprinting

Additional proteins associated with TIM-3 were identified in lysates from treated 1 × 10^6^ macrophages derived from three healthy donors. Samples were analyzed at the research and industry support facility at the National Autonomous University of Mexico (UNAM) using peptide mass fingerprinting for protein identification. Initially, acrylamide bands were excised and treated for 12 hr with a 50% methanol and 5% acetic acid (v/v) solution. Samples were washed with distilled water and incubated with a 100 mM ammonium bicarbonate solution for 15 min. Reduction was performed using 50 mM DTT for 45 min, followed by alkylation with 30 mM iodoacetamide for 2 hr. The ammonium bicarbonate wash step was repeated three times. Samples were then dehydrated to complete dryness using 100% acetonitrile. Finally, the digestion process was performed with 30 *µ*L of modified porcine trypsin from a 20 ng/*µ*L stock solution for 18 hr at 37°C.

Peptide extraction was carried out by incubating the samples with 50% acetonitrile (v/v) and 5% formic acid in a sonication bath and then evaporating them to complete dryness. Next, peptides were resuspended in 1% formic acid (20 *µ*L), concentrated, and desalted with Ziptip C18 (Merck Millipore) before being eluted in 12 *µ*L of mobile phase (97% water, 3% acetonitrile, 0.1% formic acid). Samples were analyzed using LC-MS (LC nanoACQUITY, MS Synapt G2S, Waters Corp).

## 3. Results

### 3.1. Gene Expression of GAL-9 Increases in LPS-IFN-*γ* Stimulated Macrophages

TIM-3 expression profiles have been initially identified in TH1 lymphocytes and M1 macrophages [2, 16]. As a negative regulator of T-cell activation, TIM-3 overexpression has been observed in exhausted or senescent T cells [17–19]. Although TAM with an M2 tolerogenic profile expresses TIM-3, its specific role in these cells remains unclear. To investigate the TIM-3 expression profile in M1 and M2 macrophages, we employed an in vitro experimental model using human macrophages activated by the classic or alternative pathway.

**Figure 1:**
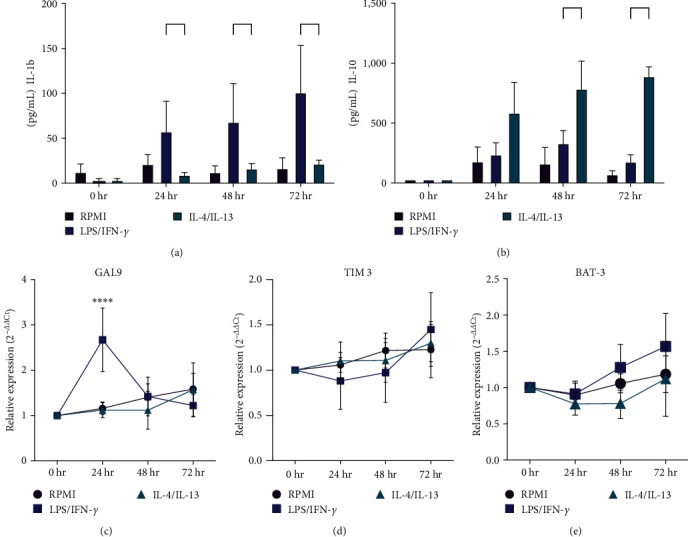
Human polarization of MDM in vitro activation. IL-1*β* (a) and IL-10 (b) were measured by ELISA in supernatants of MDM at the basal time (0), 24, 48, and 72 hr after stimulation with IFN-*γ*/LPS or IL-4/IL-13. The unstimulated condition was added as a control; (c–e) relative expression levels of GAL-9 (c), TIM-3 (d), and BAT-3 (e) were evaluated by RT-qPCR using RNA obtained from MDM at the basal time, 24, 48, and 72 hr after stimulation with IFN-*γ*/LPS or IL-4/IL-13. Data shown were represented as mean ± SD (*n* = 6)  ^*∗*^*p* < 0.05,  ^*∗∗*^*p* < 0.01,  ^*∗∗∗*^*p* < 0.001,  ^*∗∗∗∗*^*p* < 0.0001.

We evaluate characteristic cytokines IL-1*β* and IL-10 as an indicator of correct macrophage polarization. IL-1*β* levels were higher in M1 compared to M2 stimulated macrophages at 24, (M1 57.66, SD ± 33.53 vs. M2 9.20, SD ± 2.72 *p*=0.0013), 48 (M1 69.32 SD ± 42.39 vs. M2 16.17 SD ± 5.56 *p*=0.0005), and 72 hr (M1 101.4 SD ± 52.36 vs. M2 21.6 SD ± 3.92 *p*=0.0001) (Figure 1(a)). Lower production of IL-1*β* was detected in M2-stimulated macrophages.

IL-10 levels increased at 48 (M1 331.75 SD ± 264.28 vs. M2 786.681 SD ± 563.91 pg/mL *p*=0.03) and 72 hr (M1 178.53 SD ± 139.96 vs. M2 891.12 SD ± 190.66 pg/mL *p*=0.0004) in M2 macrophages (Figure 1(b)). Additionally, reduced production of IL-10 was detected in M1 subtype-induced macrophages after 24 hr of stimulation. These results confirm the functional characteristics of macrophages differentiated into M1 and M2 subtypes.

Upon obtaining mature M1 and M2 macrophages, TIM-3 expression was analyzed using qPCR and flow cytometry. Additionally, we included Galectin-9 (GAL-9) and HLA-B-associated transcript 3 (BAT3) molecules, as they are related to TIM-3 functionality. GAL-9, the most common TIM-3 ligand, exhibits a high affinity for recognizing carbohydrates present on the IgV domain of TIM-3 [20, 21]. BAT-3, an intracellular protein, interacts with the TIM-3 cytoplasmic tail, inhibiting TIM-3 signaling and effector functions in T cells [22].

TIM-3 and BAT-3 exhibited similar expression profiles in M1 (LPS/ IFN-*γ*), M2 (IL-4/IL-13), and nonstimulated macrophages across all time points (Figures 1(d) and 1(e)). GAL-9 mRNA expression increased 24 hr postexposure to IFN-*γ*/LPS, but not IL-4/IL-13 (M1 2.67 SD ± 0.7 vs. M2 1.12 SD ± 0.16-fold change) (Figure 1(c)). At the transcriptional level, no differences in TIM-3 expression were observed between M1 and M2 macrophages We analyzed TIM-3 and GAL-9 expression via flow cytometry at various time points during macrophage polarization. As previous studies reported LPS-induced downregulation of TIM-3 expression 6 hr poststimulation [6], we assessed TIM-3 and GAL-9 expression for an extended duration (72 hr). Mean-fluorescence intensity (MFI) and the percentage of positive cells with intracellular and membrane-bound TIM-3 were measured at different time points (Figures S1 and S2), using multiparametric flow cytometry (Figure 1(a)) [18, 19].

**Figure 2:**
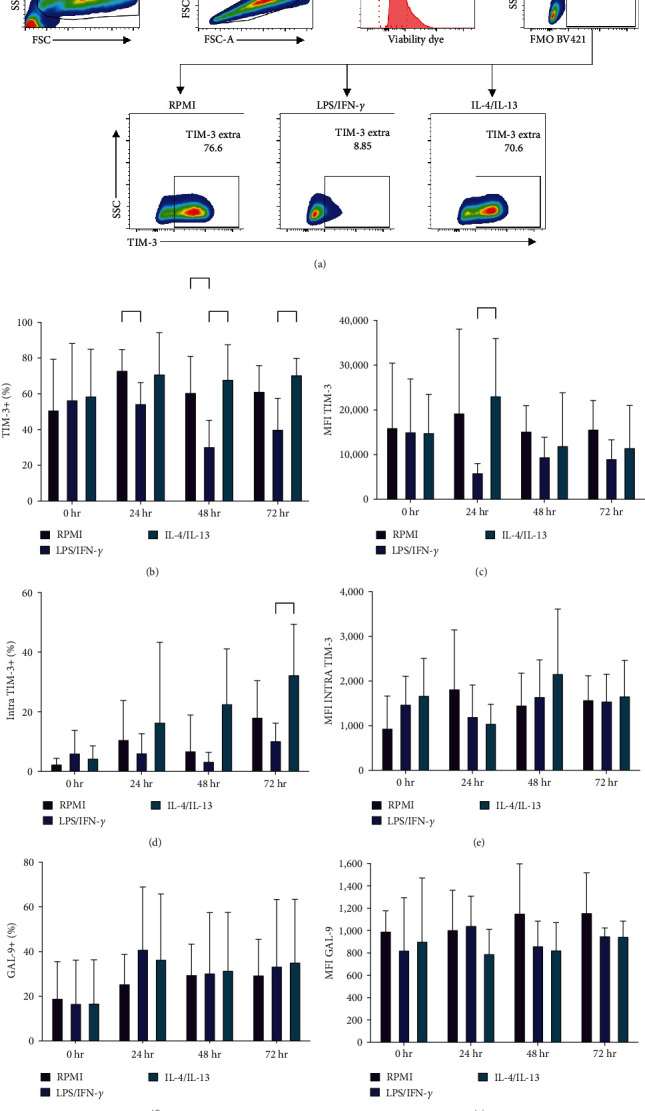
TIM-3 and GAL-9 expression on activated macrophages. TIM-3 frequency and MFI (mean fluorescence intensity) were measured on MDM by flow cytometry at basal time 24, 48, and 72 hr after stimulation with IFN-*γ*/LPS or IL-4/IL-13. (a) Representative dot plot showing analysis strategy to identify TIM-3 expression in human MDM by flow cytometry. TIM-3 was evaluated on the cell membrane (b and c) and intracellular (d and e), and GAL-9 expression was measured on the cell membrane (f and g). Data shown were represented as mean ± SD (*n* = 5)  ^*∗*^*p* < 0.05 and  ^*∗∗*^*p* < 0.01.

The percentage of TIM-3+ M1 macrophages was found to be lower compared to M2 macrophages at 48 (M1 30.68, SD ± 14.56 vs. M2 68.25 SD ± 19.23 *p*=0.0037) and 72 hr (M1 40.33 SD ± 17.19 vs. M2 70.80 SD ± 9.08 *p*=0.0029) poststimulation (Figure 2(b)). Similarly, MFI of TIM-3 in M1 macrophages was lower than in M2 macrophages (M1 6025 SD ± 1962 vs. M2 23200.2 SD ± 12782.97 *p*=0.038) at 24 hr poststimulation (Figure 2(c)). Furthermore, upon analyzing intracellular TIM-3 staining, we discovered that M1 macrophages exhibited a lower percentage of positive cells compared to M2 macrophages at 72 hr (M1 10.41 SD ± 5.73 vs. M2 32.59 SD ± 16.79 *p*=0.049) (Figure 2(d)). MFI of intracellular TIM-3 remained consistent across all time points (Figure 2(e)).

A comparable TIM-3 expression profile was observed between M2 and nonstimulated macrophages, indicating that in M1 macrophages, a downregulation mechanism is developed post-IFN-*γ* and LPS stimulation (Figure 2). GAL-9 facilitates contact with neighboring cells and adhesion to the extracellular matrix and can activate signaling cascades through receptors such as TIM-3 [24, 25]. When evaluating GAL-9, we discovered a similar frequency and MFI (Figures 2(f) and 2(g), respectively) across the three experimental conditions. This finding may suggest that GAL-9 expression is not dependent on macrophage polarization, and the absence of membrane-binding domain results in the continuous release of GAL-9 in a soluble form (sGAL-9).

### 3.2. Soluble TIM-3, GAL-9, and ADAM-10

**Figure 3:**
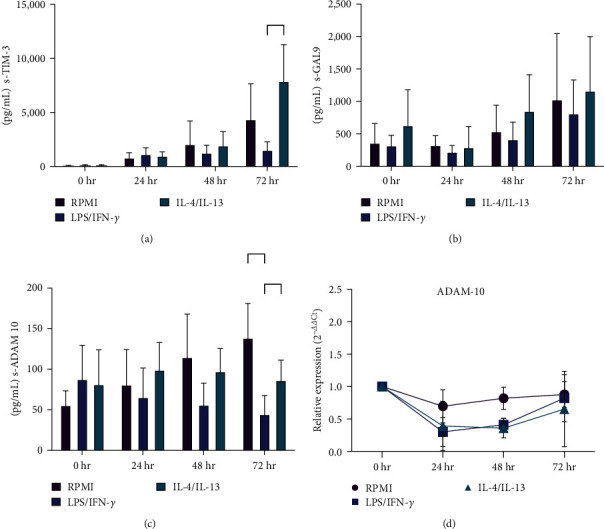
Evaluation of soluble forms of TIM-3, GAL-9, and ADAM-10 in macrophage supernatants. sTIM-3 (a), sGAL-9 (b), and sADAM-10 (c) were measured in supernatants of MDM collected at the basal time 24, 48, and 72 hr after stimulation with IFN-*γ*/LPS or IL-4/IL-13, by ELISA assay. (d) RT-PCR was performed to evaluate the relative expression of ADAM-10 on MDM stimulated with IFN-*γ*/LPS or IL-4/IL-13 at the basal time 24, 48, and 72 hr. Data shown were represented as mean ± SD (*n* = 6)  ^*∗*^*p* < 0.05 and  ^*∗∗*^*p* < 0.01.

In recent years, soluble TIM-3 (s-TIM-3) has been identified and proposed as a disease marker in viral infections [26, 27]. Using ELISA, we subsequently quantified soluble TIM-3 and GAL-9 in supernatants from stimulated macrophage cultures. Soluble TIM-3 levels remained consistent between nonstimulated and M2 macrophages at each time point, exhibiting an increase at 72 hr. However, M1 macrophages displayed a distinct profile; s-TIM-3 was significantly reduced compared to M2 macrophages at 72 hr (M1 1521, SD ± 773.89 vs. M2, 7893.87 SD ± 3384.29 pg/mL *p*=0.026) (Figure 3(a)). Supernatants were collected every 24 hr, and the highest levels of s-TIM-3 resulted from proteolytic cleavage of the cell membrane. GAL-9 levels in the supernatant were consistent across all evaluated time points (Figure 3(b)).

Clayton et al, [28], Dewitz et al. [29], and Möller-Hackbarth et al. [30] reported that ADAM-10 and ADAM-17 could mediate TIM-3 enzymatic release from the cell membrane; however, ADAM-10 has been identified as the primary enzyme responsible for this process. Therefore, ADAM-10 levels were measured in the supernatant culture from MDM. Our results showed that soluble ADAM-10 (sADAM-10) levels were consistent between nonstimulated and M2 macrophages. However, M1-macrophages exhibited lower sADAM-10 levels at 72 hr compared to nonstimulated macrophages (M1 44.36, SD ± 23.24 vs. RPMI 138.7 SD ± 42.37 pg/mL *p*=0.0038) (Figure 3(c)) and lower than M2-macrophages (M1 44.36 SD ± 23.24 vs. M2 86.37 SD ± 24.86 pg/mL *p*=0.031). mRNA was obtained from stimulated macrophages to assess ADAM-10 gene expression. At the transcriptional level, we observed similar genic expression of ADAM-10 across all conditions (Figure 3(d)).

These results suggest that the regulation of TIM-3 and ADAM-10 in activated macrophages may not occur at the gene expression level. ADAM-10 is known to maintain its proteolytic activity even in its soluble form [31]. Although existing information has primarily focused on the function of membrane-anchored ADAM-10, recent findings indicate that the soluble form of ADAM-10 (sADAM-10) exhibits different substrate specificity compared to its membrane-bound counterpart (mADAM-10) [32].

Our data reveal that soluble levels of sTIM-3 and sADAM-10 are increased in the supernatants of M2 macrophages compared to M1 macrophages. Together, these results suggest a strong likelihood that sADAM-10 specifically cleaves mTIM-3. In particular, in activated macrophages, TIM-3 and ADAM-10 appear to be regulated by enzymatic mechanisms, rather than at the gene expression level.

Previous studies with HIV patients have reported that sTIM-3 does not affect IFN-*γ* production or prevent GAL-9-induced cell death [27].

### 3.3. TIM-3 Interacts with Lymphocyte Specific Protein 1 in Human Macrophages

**Figure 4:**
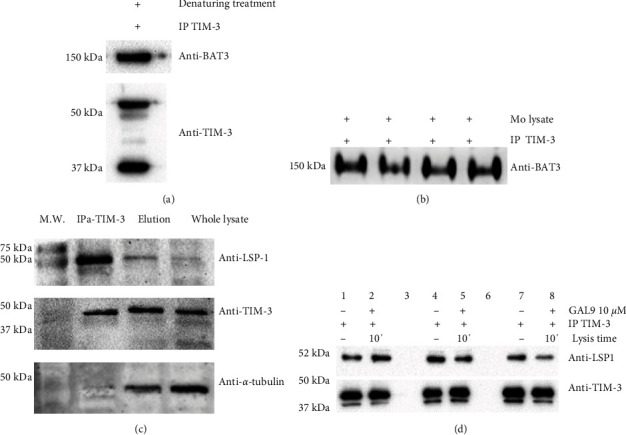
Evaluation of TIM-3 interactions through coprecipitation and western blot. (a) Immunoprecipitation was conducted on whole MDM lysates using a monoclonal anti-TIM-3 antibody. Eluted proteins underwent SDS-PAGE and subsequent western blot analysis to identify BAT-3 (upper panel in (a) and confirm the presence of TIM-3 (lower panel in a). (b) Immunoprecipitation of TIM-3 was carried out in nondenaturing conditions using whole MDM lysates from four healthy donors. Subsequent western blot analysis was performed with anti-BAT-3 antibodies to detect coprecipitated proteins. (c) Western blot analysis of LSP-1 after immunoprecipitation of TIM-3 from MDM lysates (upper blot in c). For verification, anti-TIM-3 was used for western blot analysis (middle blot in c), and alpha-tubulin was included in the western blot analysis as a loading control (bottom panel in c). Immunoprecipitation of TIM-3 and analysis of WB were performed using an anti-LSP-1 antibody. Molecular weight markers: Line 1, immunoprecipitation of TIM-3 in Line 2, eluted fraction of immunoprecipitation in Line 3, and whole lysate in Line 4 *n* = 4. (d) MDM obtained from three healthy donors were cultured in RPMI (Lanes 2, 5, and 8) or RPMI + GAL-9 10 *μ*g/mL for 10 min (Lanes 2, 4, and 6); then, control and stimulated MDM were lysed.

TIM-3 interactions with ligands such as GAL-9, phosphatidylserine, and HMGB-1 have been previously demonstrated in human and mouse models. Moreover, the intracellular tail of TIM-3 has an interaction with TCR-associated Src-family kinases such as Lck and Fyn. Additionally, BAT-3 protein interaction was established by Kuchroo V. and collaborators using an in vitro model of human lymphocytes. Furthermore, they found that BAT-3 overexpression inhibits TIM-3 functionality in T cells, a phenomenon abrogated by GAL-9/TIM-3 binding [32]. We confirmed the data reported by Kuchroo V. and collaborators through immunoprecipitation assays of TIM-3. As anticipated, we verified the TIM-3/BAT-3 interaction in human macrophages (Figures 4(a) and 4(b) and Figure S3).

In this work, our aim is to discover new interactions of TIM-3 to expand the knowledge of this receptor and its functions in macrophages. We explored new possibilities through mass fingerprinting protein identification analysis. Consequently, potential interactions were detected in TIM-3-IP samples. Numerous proteins, such as the Ig kappa chain and other structural proteins like vimentin or actin, might have been present due to the IP process (Table S2). Additionally, we observed a high-identity protein coverage (69.36%) for a protein termed lymphocyte-specific protein 1 (LSP-1), indicating a theoretical interaction with TIM-3. The LSP-1 protein is involved in cytoskeleton functionality and cell motility, and it has recently been identified as a crucial regulator in podosome formation [12, 16, 22].

We performed immunoprecipitation (IP) with an anti-TIM-3 monoclonal antibody. Following IP, SDS-PAGE, and western blot analysis were carried out using a monoclonal antibody that recognizes LSP-1 in western blotting. We identified a single band close to 50 kDa, corresponding to the LSP-1 molecular weight (Figure 4(a) upper panel and Figures S4 and S5). Subsequent analyses for TIM-3 and alpha-tubulin (*α*-Tubulin) were conducted using specific antibodies (Figure 4(a) middle panel and bottom panel, respectively).

Next, we investigated whether the TIM-3/LSP-1 interaction could be abrogated by the presence of Gal-9, as was previously demonstrated for the TIM-3/BAT-3 interaction [33]. We added Gal-9 at 10 *μ*g/mL to the culture medium of macrophages obtained from three healthy donors, and cell lysis was performed after 10 min of treatment. This interaction does not seem to be altered by the addition of Gal-9. The signal corresponding to LSP-1 is present in each sample from the different donors, both in the IP carried out with lysates cultured with RPMI and in those cultured with RPMI + Gal-9 (Figure 4(b)).

These findings suggest a novel interaction between TIM-3 and LSP-1 molecules, which may link TIM-3 to new processes and functions. Furthermore, it could help elucidate other potential mechanisms in which TIM-3 might be involved.

## 4. Discussion

In this study, we investigated the expression of TIM-3 and associated proteins during macrophage polarization to gain insight into the functionality of this receptor in myeloid cells. TIM-3 is a critical immune regulator of T-cell activation and a marker of exhausted lymphocytes. It has multiple known functions, such as blocking TCR signaling, regulating IL-12 production, and mediating phagocytosis via direct interaction with phosphatidylserine (PS). However, the expression profile, molecular interactions, and signaling of TIM-3 in myeloid cells remain incompletely understood.

We first assessed the expression of TIM-3, GAL-9, and BAT-3 by RT-PCR in human-stimulated macrophages. Prior studies have reported that various proinflammatory molecules can induce GAL-9 expression, such as IFN-*γ*, TNF-*α*, and some TLR ligands [34]. In agreement with earlier findings, we observed increased GAL-9 gene expression in macrophages stimulated with IFN-*γ*/LPS 24 hr poststimulation. TIM-3 and BAT-3 displayed similar levels of gene expression across all tested conditions. However, when we assessed protein expression via flow cytometry, we found that both intracellular and extracellular TIM-3 expression were downregulated in M1 compared to M2 macrophages at 48 and 72 hr of polarization. This supports the role of TIM-3 as a negative regulator of the inflammatory response. These findings suggest that TIM-3 expression is downregulated in M1 macrophages due to the need to remove the negative regulation exerted on macrophage activation by this receptor. This downregulation occurs at the posttranscriptional level, as we did not observe changes in TIM-3 expression at the RNA level. Our results align with observations from patients with multiple sclerosis, in which a defect in TIM-3 expression on T cells might be associated with the loss of peripheral immunoregulation of autoreactive T cells [35]. Furthermore, in a mouse model of colitis, Jiang et al. [36] demonstrated that TIM-3 inhibited the polarization process of M1 macrophages, reducing damage caused by this pro-inflammatory macrophage subtype. They also showed that TIM-3 downregulation or blockade increased the M1 pathogenic response. In contrast, in a mouse model of colitis-associated cancer and cells from colon cancer patients, it was demonstrated that TIM-3 promotes the development of tumor-supporting M2 macrophages via a STAT1-dependent pathway [37]. These data are consistent with our results, confirming that TIM-3 can modulate the pro-inflammatory profile in human macrophages. Interestingly, the soluble form of TIM-3 was present at lower levels in M1 supernatants compared to control and M2 conditions. When we evaluated TIM-3-sheddase, ADAM 10, we found reduced sADAM-10 in M1 supernatants, while M2 and nonstimulated macrophages displayed no differences. Seifert et al. [38] recently demonstrated that membrane expression of ADAM-10 is closely associated with its catalytic activity. When ADAM-10 is inactivated, it is released in exosomes or eliminated through lysosomal degradation, even in the presence of substrate [38]. However, sADAM have catalytic activity and the substrate specificity can be different between mADAM-10 and sADAM-10 [31, 32].

Despite prior information from murine models suggesting that the soluble form of TIM-3 can be produced by alternative splicing [7, 39], Kiera L. Clayton conducted a comprehensive analysis of the soluble form of TIM-3 in humans. In their study, they found that sTIM-3 present in the culture of human lymphocytes and plasma of HIV patients is generated through the shedding of TIM-3 from the cell membrane. These findings, together with our results, suggest that in M1 macrophages, lower levels of sADAM-10 are released, in consequence, less TIM-3 specific sADAM-10 is available to remove TIM-3 from the cell membrane. In nonstimulated and M2 macrophages, higher levels of sADAM-10 produce increased sTIM-3 level. This observation supports the role of TIM-3 as a negative regulator of activation in proinflammatory cells, which must be neutralized to enable proper activation of specialized cell functions in activated macrophages.

Regarding the TIM-3 ligand, GAL-9, whose expression is induced by IFN-*γ* at the mRNA level, we found the protein at similar levels in both membrane and soluble forms in all conditions. This may suggest that GAL-9 relies entirely on TIM-3 to induce negative regulation through this pathway.

Our next objective was to assess the interaction of TIM-3 with other proteins previously reported in human models. We performed immunoprecipitation (IP) to identify TIM-3-associated proteins. BAT-3, was evaluated by western blot following IP using a monoclonal antibody specific for TIM-3. As previously reported in T cells, we confirmed the interaction between BAT-3 and TIM-3. In human lymphocytes, BAT-3 association with TIM-3 prevents the interruption of TCR signaling [33]. Phosphorylation of the TIM-3 cytoplasmic tail leads to the dissociation of the TIM-3/BAT-3 complex, and TIM-3 can recruit Lck. At this point, Lck cannot continue to participate in the TCR pathway. However, is possible that this interaction can occur only in lymphocytes due the specific cell expression of Lck [40, 41].

Data from the IP assays and mass fingerprinting protein identification analysis allowed us to identify that TIM-3 interacts with LSP-1 protein in human macrophages. LSP-1 has been described as a critical regulator of actomyosin contractility in primary macrophages [42]. Other researchers have reported that LSP-1 plays a crucial role in mediating macrophage phagocytic activity through the FC-immunoglobulin gamma receptor [43]. LSP1 accumulates at sites of actin cytoskeleton remodeling and around nascent phagosomes shortly after initiating the phagocytosis process. With this prior evidence and the fact that TIM-3 coprecipitates with LSP-1, we could propose that phagocytosis triggered when TIM-3 binds to phosphatidylserine is a mechanism involving LSP-1. This hypothesis requires validation, but this data could help clarify one of the functions described for TIM-3. Another critical question arising from these results is how LSP-1 interaction or expression occurs in lymphocytes, given that these cells are not specialized phagocytes.

In conclusion, we have corroborated that TIM-3 expression in macrophages is a specific process, depending on the stimuli and functions required when classical or alternative pathways activate these cells. The regulation of TIM-3 relies more on enzymatic mechanisms than gene expression, at least in the early stages of the activation process. In addition to the BAT-3 interaction, we identified that TIM-3 interacts with the LSP-1 protein in human macrophages. These findings contribute to our understanding of TIM-3 function. However, new questions have also emerged and warrant further experimental investigation.

## Data Availability

The authors confirm that the raw data to support the conclusions of this study are included in the manuscript. The corresponding author will provide more information upon reasonable request to any qualified researcher.

## Supplementary Materials

Supplementary Materials Table S1: antibodies used in the study. Figure S1: intracellular TIM-3 evaluation in activated macrophages. Figure S2: TIM-3 evaluation in activated macrophages. Figure S3: BAT-3 interact with TIM-3. Table S2: relevant proteins identified by liquid chromatography/mass spectrometry in the eluted fraction of TIM-3 immunoprecipitation. Figure S4: TIM-3 coprecipitates with LSP-1 protein. Figure S5: TIM-3 interacts with LSP-1 protein.
