# Supplementary material for: Tim-3 Is Differentially Expressed during Cell Activation and Interacts with the LSP-1 Protein in Human Macrophages
Source: J Immunol Res. 2023 Oct 26;2023:3577334. doi: 10.1155/2023/3577334 (PMC10622183; doi:10.1155/2023/3577334)
Supplement: Supplementary Materials — Table S1: antibodies used in the study. Figure S1: intracellular TIM-3 evaluation in activated macrophages. Figure S2: TIM-3 evaluation in activated macrophages. Figure S3: BAT-3 interact with TIM-3. Table S2: relevant proteins identified by liquid chromatography/mass spectrometry in the eluted fraction of TIM-3 immunoprecipitation. Figure S4: TIM-3 coprecipitates with LSP-1 protein. Figure S5: TIM-3 interacts with LSP-1 protein. [file 3577334.f1.docx]

# Journal of Immunology Research

**Tim-3 is dynamically expressed during cell activation and interacts with the LSP-1 protein in human macrophages.**

**Supplementary Material.**

**Figure Supplementary 1.** **Intracellular TIM-3 evaluation in activated macrophages.** Representative gates from intracellular TIM-3 analysis by flow cytometry in MDM at the basal time, 24, 48, and 72 hours after stimulation with IFN-γ/LPS or IL-4/IL-13.

**Figure Supplementary 2.** **TIM-3 evaluation in activated macrophages.** Representative gates from membrane-bound TIM-3 (extra) analysis by flow cytometry in MDM at the basal time, 24, 48, and 72 hours after stimulation with IFN-γ/LPS or IL-4/IL-13.

**Supplementary table 1.** Antibodies used in the study.

| **ANTIBODY** | **BRAND** | **CLONE** | **REF. NUMBER** |
| --- | --- | --- | --- |
| Anti-mouse IgG, HRP-linked Antibody | Cell Signaling Technology | polyclonal antibody | 7076 |
| Anti-rabbit IgG, HRP-linked Antibody | Cell Signaling Technology | polyclonal antibody | 7074 |
| anti-Beta actin | Santa Cruz Biotechnology | C4 | sc-47778 |
| anti-Alpha tubulin | Genetex | polyclonal antibody | GTX112141 |
| anti-Beta actin | Genetex | polyclonal antibody | GTX109639 |
| anti-GAPDH | Genetex | polyclonal antibody | GTX100118 |
| APC anti-human CD366 (Tim-3) | BioLegend | F38-2E2 | 345012 |
| Brilliant Violet 421™ anti-human CD366 (Tim-3) Antibody | BioLegend | F38-2E2 | 345008 |
| Fyn Antibody | Cell Signaling Technology | polyclonal antibody | 4023 |
| Human/Mouse/Rat BAT3/BAG6 Antibody | R&D Systems | Polyclonal Sheep IgG | AF6438 |
| LSP1 Antibody | Cell Signaling Technology | polyclonal antibody | 3812 |
| Purified anti-Fyn | BioLegend | FYN-59 | 626502 |
| Sheep IgG Horseradish Peroxidaseconjugated Antibody | R&D Systems | polyclonal antibody | HAF016 |
| PerCP/Cyanine5.5 anti-human CD14 antibody | BioLegend | HCD14 | 325622 |
| FITC anti-human CD14 antibody | BioLegend | 63D3 | 367116 |
| PE anti-human HLA-DR antibody | BioLegend | L243 | 307606 |
| PE anti-human Galectin-9 antibody | BioLegend | 9M1-3 | 348906 |
| Human TIM-3 Allophycocyanin antibody | R&D Systems | 344823 | FAB2365A |
| LEAF™ Purified anti-human CD366 (Tim-3) antibody | BioLegend | F38-2E2 | 345004 |
| Purified anti-Lck antibody | BioLegend | LCK-01 | 628302 |

**Supplementary table 2.** Relevant proteins identified by Liquid Chromatography/Mass Spectrometry in the eluted fraction of TIM-3 immunoprecipitation.

|  | |  | |  | |  |  |
| --- | --- | --- | --- | --- | --- | --- | --- |
|  | ***Protein Match Details*** | | | | | | |
|  | ***Accession:*** | | | | *E9PBD8_HUMAN* | | |
|  | ***Description:*** | | | | *Lymphocyte specific protein 1 Fragment OS Homo sapiens GN LSP1 PE 1 SV 1* | | |
|  | ***Coverage:*** | |  | | *69.3694* | | |
|  | ***Matches:*** | |  | | *10* | | |
|  | ***Score:*** | |  | | *763.0973* | | |
|  | ***MS Score:*** | |  | | *-2.15E+09* | | |
|  | ***MS E-Value*** | | | | *-2.15E+09* | | |
|  | ***Peptide Match #*** | | ***1*** | |  | | |
|  | ***Sequence:*** | |  | | *IDQWLEQYTQAIETAGR* | | |
|  | ***Mass*** | |  | | *2021.9927* | | |
|  | ***Probability:*** | | | | *0* | | |
|  | ***Mascot Score*** | | | | *-2.15E+09* | | |
|  | ***Lad. Score*** | |  | | *-2.15E+09* | | |
|  | ***Log Lik*** | |  | | *6.4576* | | |
|  | ***Peptide Match #*** | | ***2*** | |  | | |
|  | ***Sequence:*** | |  | | *WETGEVQAQSAAK* | | |
|  | ***Mass*** | |  | | *1404.6754* | | |
|  | ***Probability:*** | | | | *0* | | |
|  | ***Mascot Score*** | | | | *-2.15E+09* | | |
|  | ***Lad. Score*** | |  | | *-2.15E+09* | | |
|  | ***Log Lik*** | |  | | *6.2771* | | |
|  | ***Peptide Match #*** | | ***3*** | |  | | |
|  | ***Sequence:*** | |  | |  |  |  |
|  | ***Mass*** | |  | | *1647.8085* | | |
|  | ***Probability:*** | | | | *0* | | |
|  | ***Mascot Score*** | | | | *-2.15E+09* | | |
|  | ***Lad. Score*** | |  | | *-2.15E+09* | | |
|  | ***Log Lik*** | |  | | *6.1115* | | |
|  | ***Peptide Match #*** | | ***4*** | |  | | |
|  | ***Sequence:*** | |  | | *QASIELPSMAVASTK* | | |
|  | ***Mass*** | |  | | *1532.799* | | |
|  | ***Probability:*** | | | | *0* | | |
|  | ***Mascot Score*** | | | | *-2.15E+09* | | |
|  | ***Lad. Score*** | |  | | *-2.15E+09* | | |
|  | ***Log Lik*** | |  | | *5.922* | | |
|  | ***Peptide Match #*** | | ***5*** | |  | | |
|  | ***Sequence:*** | |  | | *EGPGPEDTVQDNLGAAGAEEEQEEHQK* | | |
|  | ***Mass*** | |  | | *2864.2454* | | |
|  | ***Probability:*** | | | | *0* | | |
|  | ***Mascot Score*** | | | | *-2.15E+09* | | |
|  | ***Lad. Score*** | |  | | *-2.15E+09* | | |
|  | ***Log Lik*** | |  | | *5.8246* | | |
|  | ***Peptide Match #*** | | ***6*** | |  | | |
|  | ***Sequence:*** | |  | | *SPEGEQEDRPGLHAYEKEDSDEVHLEELSLSK* | | |
|  | ***Mass*** | |  | | *3652.6885* | | |
|  | ***Probability:*** | | | | *0* | | |
|  | ***Mascot Score*** | | | | *-2.15E+09* | | |
|  | ***Lad. Score*** | |  | | *-2.15E+09* | | |
|  | ***Log Lik*** | |  | | *5.7233* | | |
|  | ***Peptide Match #*** | | ***7*** | |  | | |
|  | ***Sequence:*** | |  | | *QQHEGAQGALDSGEPPQCR* | | |
|  | ***Mass*** | |  | | *2064.9153* | | |
|  | ***Probability:*** | | | | *0* | | |
|  | ***Mascot Score*** | | | | *-2.15E+09* | | |
|  | ***Lad. Score*** | |  | | *-2.15E+09* | | |
|  | ***Log Lik*** | |  | | *5.4285* | | |
|  | ***Peptide Match #*** | | ***8*** | |  | | |
|  | ***Sequence:*** | |  | | *MLLSLKPSEAPELDEDEGFGDWSQRPEQR* | | |
|  | ***Mass*** | |  | | *3359.585* | | |
|  | ***Probability:*** | | | | *0* | | |
|  | ***Mascot Score*** | | | | *-2.15E+09* | | |
|  | ***Lad. Score*** | |  | | *-2.15E+09* | | |
|  | ***Log Lik*** | |  | | *5.0602* | | |
|  | ***Peptide Match #*** | | ***9*** | |  | | |
|  | ***Sequence:*** | |  | | *IDQWLEQYTQAIETAGR* | | |
|  | ***Mass*** | |  | | *2003.982* | | |
|  | ***Probability:*** | | | | *0* | | |
|  | ***Mascot Score*** | | | | *-2.15E+09* | | |
|  | ***Lad. Score*** | |  | | *-2.15E+09* | | |
|  | ***Log Lik*** | |  | | *0* | | |
|  | ***Peptide Match #*** | | ***10*** | |  | | |
|  | ***Sequence:*** | |  | | *WETGEVQAQSAAK* | | |
|  | ***Mass*** | |  | | *1387.6488* | | |
|  | ***Probability:*** | | | | *0* | | |
|  | ***Mascot Score*** | | | | *-2.15E+09* | | |
|  | ***Lad. Score*** | |  | | *-2.15E+09* | | |
|  | ***Log Lik*** | |  | | *0* | | |
|  |  | | | | | | |
|  | ***Accession:*** | | | | *A0A173GMX0_HUMAN* | | |
|  | ***Description:*** | | | | *Actin alpha cardiac muscle 1 Fragment OS Homo sapiens GN ACTC1 PE 4 SV 1* | | |
|  | ***Coverage:*** | |  | | *25.3968* | | |
|  | ***Matches:*** | |  | | *2* | | |
|  | ***Score:*** | |  | | *5346.083* | | |
|  | ***MS Score:*** | |  | | *-2.15E+09* | | |
|  | ***MS E-Value*** | | | | *-2.15E+09* | | |
|  | ***Peptide Match #*** | | ***1*** | |  | | |
|  | ***Sequence:*** | |  | | *SYELPDGQVITIGNER* | | |
|  | ***Mass*** | |  | | *1790.8918* | | |
|  | ***Probability:*** | | | | *0* | | |
|  | ***Mascot Score*** | | | | *-2.15E+09* | | |
|  | ***Lad. Score*** | |  | | *-2.15E+09* | | |
|  | ***Log Lik*** | |  | | *8.0654* | | |
|  | ***Peptide Match #*** | | ***2*** | |  | | |
|  | ***Sequence:*** | |  | | *SYELPDGQVI* | | |
|  | ***Mass*** | |  | | *1102.5415* | | |
|  | ***Probability:*** | | | | *0* | | |
|  | ***Mascot Score*** | | | | *-2.15E+09* | | |
|  | ***Lad. Score*** | |  | | *-2.15E+09* | | |
|  | ***Log Lik*** | |  | | *0* | | |
|  |  | | | | | | |
|  | ***Accession:*** | | | | *VIME_HUMAN* | | |
|  | ***Description:*** | | | | *Vimentin OS Homo sapiens GN VIM PE 1 SV 4* | | |
|  | ***Coverage:*** | |  | | *38.412* | | |
|  | ***Matches:*** | |  | | *33* | | |
|  | ***Score:*** | |  | | *18372.91* | | |
|  | ***MS Score:*** | |  | | *-2.15E+09* | | |
|  | ***MS E-Value*** | | | | *-2.15E+09* | | |
|  | ***Peptide Match #*** | | ***1*** | |  | | |
|  | ***Sequence:*** | |  | | *EEAENTLQSFR* | | |
|  | ***Mass*** | |  | | *1323.6176* | | |
|  | ***Probability:*** | | | | *0* | | |
|  | ***Mascot Score*** | | | | *-2.15E+09* | | |
|  | ***Lad. Score*** | |  | | *-2.15E+09* | | |
|  | ***Log Lik*** | |  | | *8.9347* | | |
|  | ***Peptide Match #*** | | ***2*** | |  | | |
|  | ***Sequence:*** | |  | | *DGQVINETSQHHDDLE* | | |
|  | ***Mass*** | |  | | *1836.7996* | | |
|  | ***Probability:*** | | | | *0* | | |
|  | ***Mascot Score*** | | | | *-2.15E+09* | | |
|  | ***Lad. Score*** | |  | | *-2.15E+09* | | |
|  | ***Log Lik*** | |  | | *8.9335* | | |
|  | ***Peptide Match #*** | | ***3*** | |  | | |
|  | ***Sequence:*** | |  | | *SLYASSPGGVYATR* | | |
|  | ***Mass*** | |  | | *1428.7118* | | |
|  | ***Probability:*** | | | | *0* | | |
|  | ***Mascot Score*** | | | | *-2.15E+09* | | |
|  | ***Lad. Score*** | |  | | *-2.15E+09* | | |
|  | ***Log Lik*** | |  | | *8.7929* | | |
|  | ***Peptide Match #*** | | ***4*** | |  | | |
|  | ***Sequence:*** | |  | | *LLQDSVDFSLADAINTEFK* | | |
|  | ***Mass*** | |  | | *2126.0652* | | |
|  | ***Probability:*** | | | | *0* | | |
|  | ***Mascot Score*** | | | | *-2.15E+09* | | |
|  | ***Lad. Score*** | |  | | *-2.15E+09* | | |
|  | ***Log Lik*** | |  | | *8.7357* | | |
|  | ***Peptide Match #*** | | ***5*** | |  | | |
|  | ***Sequence:*** | |  | | *ETNLDSLPLVDTHSK* | | |
|  | ***Mass*** | |  | | *1668.8439* | | |
|  | ***Probability:*** | | | | *0* | | |
|  | ***Mascot Score*** | | | | *-2.15E+09* | | |
|  | ***Lad. Score*** | |  | | *-2.15E+09* | | |
|  | ***Log Lik*** | |  | | *8.7077* | | |
|  | ***Peptide Match #*** | | ***6*** | |  | | |
|  | ***Sequence:*** | |  | | *FADLSEAANR* | | |
|  | ***Mass*** | |  | | *1093.5273* | | |
|  | ***Probability:*** | | | | *0* | | |
|  | ***Mascot Score*** | | | | *-2.15E+09* | | |
|  | ***Lad. Score*** | |  | | *-2.15E+09* | | |
|  | ***Log Lik*** | |  | | *8.6679* | | |
|  | ***Peptide Match #*** | | ***7*** | |  | | |
|  | ***Sequence:*** | |  | | *QVQSLTCEVDALK* | | |
|  | ***Mass*** | |  | | *1490.752* | | |
|  | ***Probability:*** | | | | *0* | | |
|  | ***Mascot Score*** | | | | *-2.15E+09* | | |
|  | ***Lad. Score*** | |  | | *-2.15E+09* | | |
|  | ***Log Lik*** | |  | | *8.6108* | | |
|  | ***Peptide Match #*** | | ***8*** | |  | | |
|  | ***Sequence:*** | |  | | *VELQELNDR* | | |
|  | ***Mass*** | |  | | *1115.5691* | | |
|  | ***Probability:*** | | | | *0* | | |
|  | ***Mascot Score*** | | | | *-2.15E+09* | | |
|  | ***Lad. Score*** | |  | | *-2.15E+09* | | |
|  | ***Log Lik*** | |  | | *8.5632* | | |
|  | ***Peptide Match #*** | | ***9*** | |  | | |
|  | ***Sequence:*** | |  | | *ISLPLPNFSSLNLR* | | |
|  | ***Mass*** | |  | | *1570.8951* | | |
|  | ***Probability:*** | | | | *0* | | |
|  | ***Mascot Score*** | | | | *-2.15E+09* | | |
|  | ***Lad. Score*** | |  | | *-2.15E+09* | | |
|  | ***Log Lik*** | |  | | *8.5615* | | |
|  | ***Peptide Match #*** | | ***10*** | |  | | |
|  | ***Sequence:*** | |  | | *EYQDLLNVK* | | |
|  | ***Mass*** | |  | | *1121.5837* | | |
|  | ***Probability:*** | | | | *0* | | |
|  | ***Mascot Score*** | | | | *-2.15E+09* | | |
|  | ***Lad. Score*** | |  | | *-2.15E+09* | | |
|  | ***Log Lik*** | |  | | *8.5484* | | |
|  | ***Peptide Match #*** | | ***11*** | |  | | |
|  | ***Sequence:*** | |  | | *TYSLGSALRPSTSR* | | |
|  | ***Mass*** | |  | | *1495.7863* | | |
|  | ***Probability:*** | | | | *0* | | |
|  | ***Mascot Score*** | | | | *-2.15E+09* | | |
|  | ***Lad. Score*** | |  | | *-2.15E+09* | | |
|  | ***Log Lik*** | |  | | *8.2893* | | |
|  | ***Peptide Match #*** | | ***12*** | |  | | |
|  | ***Sequence:*** | |  | | *TNEKVELQELNDR* | | |
|  | ***Mass*** | |  | | *1587.7972* | | |
|  | ***Probability:*** | | | | *0* | | |
|  | ***Mascot Score*** | | | | *-2.15E+09* | | |
|  | ***Lad. Score*** | |  | | *-2.15E+09* | | |
|  | ***Log Lik*** | |  | | *8.2326* | | |
|  | ***Peptide Match #*** | | ***13*** | |  | | |
|  | ***Sequence:*** | |  | | *FANYIDK* | | |
|  | ***Mass*** | |  | | *870.4356* | | |
|  | ***Probability:*** | | | | *0* | | |
|  | ***Mascot Score*** | | | | *-2.15E+09* | | |
|  | ***Lad. Score*** | |  | | *-2.15E+09* | | |
|  | ***Log Lik*** | |  | | *8.2103* | | |
|  | ***Peptide Match #*** | | ***14*** | |  | | |
|  | ***Sequence:*** | |  | | *NLQEAEEWYK* | | |
|  | ***Mass*** | |  | | *1309.606* | | |
|  | ***Probability:*** | | | | *0* | | |
|  | ***Mascot Score*** | | | | *-2.15E+09* | | |
|  | ***Lad. Score*** | |  | | *-2.15E+09* | | |
|  | ***Log Lik*** | |  | | *8.1113* | | |
|  | ***Peptide Match #*** | | ***15*** | |  | | |
|  | ***Sequence:*** | |  | | *VEVERDNLAEDIMR* | | |
|  | ***Mass*** | |  | | *1688.8271* | | |
|  | ***Probability:*** | | | | *0* | | |
|  | ***Mascot Score*** | | | | *-2.15E+09* | | |
|  | ***Lad. Score*** | |  | | *-2.15E+09* | | |
|  | ***Log Lik*** | |  | | *6.6662* | | |
|  | ***Peptide Match #*** | | ***16*** | |  | | |
|  | ***Sequence:*** | |  | | *PGGVYATR* | | |
|  | ***Mass*** | |  | | *820.4312* | | |
|  | ***Probability:*** | | | | *0* | | |
|  | ***Mascot Score*** | | | | *-2.15E+09* | | |
|  | ***Lad. Score*** | |  | | *-2.15E+09* | | |
|  | ***Log Lik*** | |  | | *0* | | |
|  | ***Peptide Match #*** | | ***17*** | |  | | |
|  | ***Sequence:*** | |  | | *LLQDSVDFSLADAINTEFK* | | |
|  | ***Mass*** | |  | | *2108.0547* | | |
|  | ***Probability:*** | | | | *0* | | |
|  | ***Mascot Score*** | | | | *-2.15E+09* | | |
|  | ***Lad. Score*** | |  | | *-2.15E+09* | | |
|  | ***Log Lik*** | |  | | *0* | | |
|  | ***Peptide Match #*** | | ***18*** | |  | | |
|  | ***Sequence:*** | |  | | *VELQE* | | |
|  | ***Mass*** | |  | | *581.293* | | |
|  | ***Probability:*** | | | | *0* | | |
|  | ***Mascot Score*** | | | | *-2.15E+09* | | |
|  | ***Lad. Score*** | |  | | *-2.15E+09* | | |
|  | ***Log Lik*** | |  | | *0* | | |
|  | ***Peptide Match #*** | | ***19*** | |  | | |
|  | ***Sequence:*** | |  | | *FANYIDK* | | |
|  | ***Mass*** | |  | | *853.409* | | |
|  | ***Probability:*** | | | | *0* | | |
|  | ***Mascot Score*** | | | | *-2.15E+09* | | |
|  | ***Lad. Score*** | |  | | *-2.15E+09* | | |
|  | ***Log Lik*** | |  | | *0* | | |
|  | ***Peptide Match #*** | | ***20*** | |  | | |
|  | ***Sequence:*** | |  | | *NYIDK* | | |
|  | ***Mass*** | |  | | *652.3301* | | |
|  | ***Probability:*** | | | | *0* | | |
|  | ***Mascot Score*** | | | | *-2.15E+09* | | |
|  | ***Lad. Score*** | |  | | *-2.15E+09* | | |
|  | ***Log Lik*** | |  | | *0* | | |
|  | ***Peptide Match #*** | | ***21*** | |  | | |
|  | ***Sequence:*** | |  | | *QSFR* | | |
|  | ***Mass*** | |  | | *519.2674* | | |
|  | ***Probability:*** | | | | *0* | | |
|  | ***Mascot Score*** | | | | *-2.15E+09* | | |
|  | ***Lad. Score*** | |  | | *-2.15E+09* | | |
|  | ***Log Lik*** | |  | | *0* | | |
|  | ***Peptide Match #*** | | ***22*** | |  | | |
|  | ***Sequence:*** | |  | | *NLQE* | | |
|  | ***Mass*** | |  | | *485.2354* | | |
|  | ***Probability:*** | | | | *0* | | |
|  | ***Mascot Score*** | | | | *-2.15E+09* | | |
|  | ***Lad. Score*** | |  | | *-2.15E+09* | | |
|  | ***Log Lik*** | |  | | *0* | | |
|  | ***Peptide Match #*** | | ***23*** | |  | | |
|  | ***Sequence:*** | |  | | *QEAEEWYK* | | |
|  | ***Mass*** | |  | | *1082.4789* | | |
|  | ***Probability:*** | | | | *0* | | |
|  | ***Mascot Score*** | | | | *-2.15E+09* | | |
|  | ***Lad. Score*** | |  | | *-2.15E+09* | | |
|  | ***Log Lik*** | |  | | *0* | | |
|  | ***Peptide Match #*** | | ***24*** | |  | | |
|  | ***Sequence:*** | |  | | *EAEEWYK* | | |
|  | ***Mass*** | |  | | *954.4203* | | |
|  | ***Probability:*** | | | | *0* | | |
|  | ***Mascot Score*** | | | | *-2.15E+09* | | |
|  | ***Lad. Score*** | |  | | *-2.15E+09* | | |
|  | ***Log Lik*** | |  | | *0* | | |
|  | ***Peptide Match #*** | | ***25*** | |  | | |
|  | ***Sequence:*** | |  | | *QDLLNVK* | | |
|  | ***Mass*** | |  | | *829.4778* | | |
|  | ***Probability:*** | | | | *0* | | |
|  | ***Mascot Score*** | | | | *-2.15E+09* | | |
|  | ***Lad. Score*** | |  | | *-2.15E+09* | | |
|  | ***Log Lik*** | |  | | *0* | | |
|  | ***Peptide Match #*** | | ***26*** | |  | | |
|  | ***Sequence:*** | |  | | *ISLPLPNFSSLNLR* | | |
|  | ***Mass*** | |  | | *1552.8846* | | |
|  | ***Probability:*** | | | | *0* | | |
|  | ***Mascot Score*** | | | | *-2.15E+09* | | |
|  | ***Lad. Score*** | |  | | *-2.15E+09* | | |
|  | ***Log Lik*** | |  | | *0* | | |
|  | ***Peptide Match #*** | | ***27*** | |  | | |
|  | ***Sequence:*** | |  | | *SSLNLR* | | |
|  | ***Mass*** | |  | | *689.3941* | | |
|  | ***Probability:*** | | | | *0* | | |
|  | ***Mascot Score*** | | | | *-2.15E+09* | | |
|  | ***Lad. Score*** | |  | | *-2.15E+09* | | |
|  | ***Log Lik*** | |  | | *0* | | |
|  | ***Peptide Match #*** | | ***28*** | |  | | |
|  | ***Sequence:*** | |  | | *SLNLR* | | |
|  | ***Mass*** | |  | | *602.362* | | |
|  | ***Probability:*** | | | | *0* | | |
|  | ***Mascot Score*** | | | | *-2.15E+09* | | |
|  | ***Lad. Score*** | |  | | *-2.15E+09* | | |
|  | ***Log Lik*** | |  | | *0* | | |
|  | ***Peptide Match #*** | | ***29*** | |  | | |
|  | ***Sequence:*** | |  | | *LNLR* | | |
|  | ***Mass*** | |  | | *515.33* | | |
|  | ***Probability:*** | | | | *0* | | |
|  | ***Mascot Score*** | | | | *-2.15E+09* | | |
|  | ***Lad. Score*** | |  | | *-2.15E+09* | | |
|  | ***Log Lik*** | |  | | *0* | | |
|  | ***Peptide Match #*** | | ***30*** | |  | | |
|  | ***Sequence:*** | |  | | *NLR* | | |
|  | ***Mass*** | |  | | *402.2459* | | |
|  | ***Probability:*** | | | | *0* | | |
|  | ***Mascot Score*** | | | | *-2.15E+09* | | |
|  | ***Lad. Score*** | |  | | *-2.15E+09* | | |
|  | ***Log Lik*** | |  | | *0* | | |
|  | ***Peptide Match #*** | | ***31*** | |  | | |
|  | ***Sequence:*** | |  | | *VDTHSK* | | |
|  | ***Mass*** | |  | | *668.3362* | | |
|  | ***Probability:*** | | | | *0* | | |
|  | ***Mascot Score*** | | | | *-2.15E+09* | | |
|  | ***Lad. Score*** | |  | | *-2.15E+09* | | |
|  | ***Log Lik*** | |  | | *0* | | |
|  | ***Peptide Match #*** | | ***32*** | |  | | |
|  | ***Sequence:*** | |  | | *VDTHSK* | | |
|  | ***Mass*** | |  | | *686.3468* | | |
|  | ***Probability:*** | | | | *0* | | |
|  | ***Mascot Score*** | | | | *-2.15E+09* | | |
|  | ***Lad. Score*** | |  | | *-2.15E+09* | | |
|  | ***Log Lik*** | |  | | *0* | | |
|  | ***Peptide Match #*** | | ***33*** | |  | | |
|  | ***Sequence:*** | |  | | *INETSQHHDDLE* | | |
|  | ***Mass*** | |  | | *1419.6135* | | |
|  | ***Probability:*** | | | | *0* | | |
|  | ***Mascot Score*** | | | | *-2.15E+09* | | |
|  | ***Lad. Score*** | |  | | *-2.15E+09* | | |
|  | ***Log Lik*** | |  | | *0* | | |
|  |  | | | | | | |
|  | ***Accession:*** | | | | *IGKC_MOUSE* | | |
|  | ***Description:*** | | | | *Ig kappa chain C region OS Mus musculus PE 1 SV 1* | | |
|  | ***Coverage:*** | |  | | *63.2076* | | |
|  | ***Matches:*** | |  | | *7* | | |
|  | ***Score:*** | |  | | *2921.085* | | |
|  | ***MSScore:*** | |  | | *-2.15E+09* | | |
|  | ***MS E-Value*** | | | | *-2.15E+09* | | |
|  | ***Peptide Match #*** | | ***1*** | |  | | |
|  | ***Sequence:*** | |  | | *QNGVLNSWTDQDSK* | | |
|  | ***Mass*** | |  | | *1591.7347* | | |
|  | ***Probability:*** | | | | *0* | | |
|  | ***Mascot Score*** | | | | *-2.15E+09* | | |
|  | ***Lad. Score*** | |  | | *-2.15E+09* | | |
|  | ***Log Lik*** | |  | | *7.7487* | | |
|  | ***Peptide Match #*** | | ***2*** | |  | | |
|  | ***Sequence:*** | |  | | *DSTYSMSSTLTLTKDEYER* | | |
|  | ***Mass*** | |  | | *2227.007* | | |
|  | ***Probability:*** | | | | *0* | | |
|  | ***Mascot Score*** | | | | *-2.15E+09* | | |
|  | ***Lad. Score*** | |  | | *-2.15E+09* | | |
|  | ***Log Lik*** | |  | | *6.4572* | | |
|  | ***Peptide Match #*** | | ***3*** | |  | | |
|  | ***Sequence:*** | |  | | *ADAAPTVSIFPPSSEQLTSGGASVVCFLNNFYPK* | |  |
|  | ***Mass*** | |  | | *3571.7415* | | |
|  | ***Probability:*** | | | | *0* | | |
|  | ***Mascot Score*** | | | | *-2.15E+09* | | |
|  | ***Lad. Score*** | |  | | *-2.15E+09* | | |
|  | ***Log Lik*** | |  | | *6.2821* | | |
|  | ***Peptide Match #*** | | ***4*** | |  | | |
|  | ***Sequence:*** | |  | | *QNGVL* | | |
|  | ***Mass*** | |  | | *495.2562* | | |
|  | ***Probability:*** | | | | *0* | | |
|  | ***Mascot Score*** | | | | *-2.15E+09* | | |
|  | ***Lad. Score*** | |  | | *-2.15E+09* | | |
|  | ***Log Lik*** | |  | | *0* | | |
|  | ***Peptide Match #*** | | ***5*** | |  | | |
|  | ***Sequence:*** | |  | | *NGVLNSWTDQDSK* | | |
|  | ***Mass*** | |  | | *1463.6761* | | |
|  | ***Probability:*** | | | | *0* | | |
|  | ***Mascot Score*** | | | | *-2.15E+09* | | |
|  | ***Lad. Score*** | |  | | *-2.15E+09* | | |
|  | ***Log Lik*** | |  | | *0* | | |
|  | ***Peptide Match #*** | | ***6*** | |  | | |
|  | ***Sequence:*** | |  | | *WTDQDSK* | | |
|  | ***Mass*** | |  | | *879.3843* | | |
|  | ***Probability:*** | | | | *0* | | |
|  | ***Mascot Score*** | | | | *-2.15E+09* | | |
|  | ***Lad. Score*** | |  | | *-2.15E+09* | | |
|  | ***Log Lik*** | |  | | *0* | | |
|  | ***Peptide Match #*** | | ***7*** | |  | | |
|  | ***Sequence:*** | |  | | *KDEYER* | | |
|  | ***Mass*** | |  | | *821.3788* | | |
|  | ***Probability:*** | | | | *0* | | |
|  | ***Mascot Score*** | | | | *-2.15E+09* | | |
|  | ***Lad. Score*** | |  | | *-2.15E+09* | | |
|  | ***Log Lik*** | |  | | *0* | | |

*This report was obtained from the MS spectrum, showing each CURATED protein and its SCORE and MASCOT SCORE along with a coverage percentage. Also, displayed peptides are sorted by decreasing SCORE and MASCOT SCORE.*

**Figure Supplementary 3.** **BAT-3 interact with TIM-3.** Representative western blots of immunoprecipitation (IP) using an anti-TIM-3 antibody. IP elution analyzed by western blot (WB), in denaturing conditions with anti-BAT-3 antibody (A), following to a striping step and verification with a different anti-TIM-3 antibody (B). Macrophages of four healthy donors were lysates to perform IP with anti-TIM-3 and electrophoresis in non-denaturing conditions (lines 2-5), to identify TIM-3/BAT-3 interaction in WB using an anti-BAT-3 antibody.

**Supplementary Figure 4: TIM-3 Co-precipitates with LSP-1 Protein.** (A) Western blot analysis of LSP-1 after immunoprecipitation of TIM-3 from MDM lysates. For verification, anti-TIM-3 was used for Western blot analysis. (B) Alpha-tubulin was included in the Western blot analysis as a loading control. (C) Molecular weight markers in Line 1, Immunoprecipitation of TIM-3 in Line 2, eluted fraction of immunoprecipitation in Line 3, and whole lysate in Line 4. (Representative blot of a total n=4).

* The band that appears in line 1 near 25 kDa may correspond to a recognition of the light chain of the antibody used for immunoprecipitation.

**Figure Supplementary 5. TIM-3 interacts with LSP-1 protein.** Representative blots of IP using an anti-TIM-3 antibody in lysates from human macrophages of three healthy donors. The TIM-3/LSP-1 interaction was evaluated using an anti-LSP-1 in samples obtained from macrophages stimulated with GAL9 10 μM for 10 minutes or unstimulated cells (A). Verification for TIM-3 was performed after a stripping step (B).
